# Evaluating the integrated antimicrobial stewardship system of China by the assessment tool of WHO

**DOI:** 10.1002/hcs2.16

**Published:** 2022-09-13

**Authors:** Yonghong Xiao, Wei Yu, Tingting Xiao, Ping Shen

**Affiliations:** ^1^ State Key Laboratory for Diagnosis and Treatment of Infectious Diseases, National Clinical Research Center for Infectious Diseases, National Medical Center of Infectious Diseases, The First affiliated Hospital, School of Medicine Zhejiang University Hangzhou China

**Keywords:** antimicrobial stewardship, China, evaluation

## Abstract

**Objective:**

To fully understand the overall system and implementation of antimicrobial stewardship (AMS) at different levels in China, an evaluation according to the integrated AMS evaluation tool developed by World Health Organization (WHO) was conducted.

**Methods:**

A comprehensive search on the AMS relevant government policies, regulations, scientific research results, public and social activities was conducted, and the implementation of AMS strategies in national, subnational and hospital level were evaluated by the standards of the WHO.

**Results:**

The results shew that the system construction, technical infrastructure and actions of AMS in China at the national level is relatively satisfied, but the AMS system needs to be further strengthened at the subnational and the medical institutional level, especially the integration of multidisciplinary teams and relevant departments; the implementation of professional intervention strategies, national education and publicity, professional education and training are relatively weak. AMS implementation is mainly promoted at the national level, and AMS in primary medical institutions is almost missing. It is necessary to carry out AMS at the provincial level and medical institutions as soon as possible. The focus is to establish a professional AMS team, prepare AMS guideline, implement AMS strategy, raise public awareness and ensure the long‐term and sustainable development of AMS in the country.

**Conclusion:**

The Chinese government has established a system for AMS, and implemented a multisectoral coordinative mechanism. However, at the subnational and district levels, an AMS system and practice should be set up soon to promote the rational use of antibiotics.

## Graphical abstract

This figure indicated that the system construction, technical infrastructure, and actions of antimicrobial stewardship (AMS) in China at the national level are relatively satisfied, but the AMS system needs to be further strengthened at the subnational and the medical institutional level, especially in the building of multidisciplinary teams, the implementation of professional intervention strategies, professional education, and publicity.

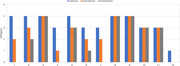

**Abbreviations**
AMR: antimicrobial resistance
AMS: antimicrobial stewardship
ARCU: administrative regulations for the clinical use of antibiotics
ARHI: administrative regulations of hospital infection
CCDC: Chinese Center for Disease Control and Prevention
CMA: Chinese Medical Association
CMDA: Chinese Medical Doctor Association
CVMA: Chinese Veterinary Medical Association
DAA: Drug Administrative Act
EML: essential medicine list
GMP: good manufacture practice
GSP: good sale practice
IPC: infection prevent and control
MCT: Ministry of Culture and Tourism
MEE: Ministry of Ecology and Environment
MIIT: Ministry of Industry and Information Technology
MOA: Ministry of Agriculture
MOE: Ministry of Education
MOHURD: Ministry of Housing and Urban–Rural Development
MOST: Ministry of Science and Technology
NAP: National Action Plan for the Containment of AMR
NEML: National Essential Medicine List
NGA: National Guideline for Antimicrobial Therapy
NHC: Chinese National Health Commission
NHC: Chinese National Health Commission
NHSA: National Healthcare Security Administration
NMPA: National Medical Product Administration
NRTA: National Radio and Television Administration
NSNC: National Surveillance Network for the Consumption of Antibiotics in Hospital
NSNR: National Surveillance Network for Bacterial Resistance
PCUA: principles for clinical use of antimicrobial agents
PHC: Provincial Health Commission
WAAW: World Antibiotic Awareness Week
WHO: World Health Organization

## 1 BACKGROUND

Antimicrobial resistance (AMR) has become a serious public health crisis threatening the world. According to a report by the British Prime Minister‐appointed Antimicrobial Resistance Review Group, if the AMR was not well‐controlled, 10 million people worldwide will die from drug‐resistant bacterial infections annually by 2050, and the cumulative economic loss will reach US$100 trillion [1]. By the prediction of the Drug Resistance Coordinator, 1.27 million patients worldwide would die directly from drug‐resistant bacterial infections in 2019 [2]. AMR containment is not only a responsibility in the field of health but also needs political attention and multisectoral joint efforts. At the 2015 summit, the G7 group decided to take active measures to tackle the increasingly severe AMR by increasing investment and scientific research. At the G20 summit in 2016 and 2017, AMR control was also included in the Communique reached by member countries. The World Health Organization (WHO) formulated the Global Action Plan on Antimicrobial Resistance in 2015 and appealed to the member countries to formulate their own national action plans for AMR control adapting to their own conditions [3]. What is more noteworthy is that in 2016, the UN General Assembly held a high‐level general debate on AMR, which is the fourth time in the history of the UN General Assembly to discuss health issues [4]. The WHO action plan proposes five strategies, which include improving awareness and understanding of AMR through effective communication, education, and training; strengthening the knowledge and evidence base through surveillance and research; reducing the incidence of infection through effective sanitation, hygiene, and infection prevention measures; optimizing the use of antimicrobial medicines in human and animal health; developing the economic case for sustainable investment that takes account of the needs of all countries and to increase investment in new medicines, diagnostic tools, vaccines, and other interventions [3]. To facilitate and support the implementation of antimicrobial stewardship (AMS), WHO prepared “AMS programs in health‐care facilities in low‐ and middle‐income countries (a WHO practical toolkit),” including AMS initiation, structure, resources, strategies, and step‐by‐step approaches, providing guidance to the Member States [5].

China is a major consumer of antibiotics, and AMR is also very prominent. To tackle AMR, the health authority of China has issued many administrative regulations and technical specifications during the past 20 years. In 2011, the Chinese National Health Commission (NHC) initiated a national special campaign for AMS, which continues right up to the present and has achieved remarkable results, and the “administrative regulations for the clinical use of antibiotics” (ARCU) was issued in 2012 [6, 7]. In 2016, the NHC, joining hands with 13 other ministries, issued the National Action Plan (NAP) to contain AMR (2016–2020) [8]. The NAP specifies that AMR control is a systematic project, involving a wide range of fields and requiring the active cooperation of multiple disciplines to achieve success.

To determine the achievements and find what needs to be improved in AMS implementation, we evaluated the Chinese AMS system at the national, subnational, and institutional levels by WHO Periodic National and Health‐Care Facility Assessment Tools [9].

## 2 METHODS

### 2.1 Data retrieval and search

To collect the overall information and various activities of AMS in China (especially in the recent 10 years), a comprehensive search on the AMS relevant government policies, regulations, scientific research results, and public and social activities was conducted. The specific search strategies are as follows.

#### 2.1.1 National and subnational policies retrieval and search

Policy documents, announcements, and reports issued by the China government were searched and retrieved by keyword searches on the national government's official websites. On each website, AMR and AMS policy keywords derived from direct translation and context translation were applied in the search. Keywords used were “antimicrobial/antibiotic resistance” OR “antimicrobial/antibiotic use,” AND “surveillance,” “antimicrobial stewardship,” “infection prevention and control,” “education,” “public awareness,” “medicines regulation,” “rational use,” “management/administration.” The major websites included the authorities of the central government, health, drug, market, education, information, and industry. Zhejiang province was selected as a representative for the subnational AMS implementation.

#### 2.1.2 Scientific publication retrieval and search

We retrieved PubMed (https://pubmed.ncbi.nlm.nih.gov) and Wanfang data (https://www.wanfangdata.com.cn/index.html) (the most comprehensive Chinese science and technology publication database) for both English and Chinese literature, and the following keywords were used: “antimicrobial/antibiotic resistance” OR “antimicrobial/antibiotic use,” AND “surveillance,” “antimicrobial stewardship,” “infection prevention and control,” “education,” “public awareness,” “medicines regulation,” “rational use,” “management/administration,” “study,” and “investigation.” The word “China” OR “Chinese” was added to the PubMed search equation, and Chinese words were used in the Wanfang data search.

#### 2.1.3 Social and public activities retrieval and search

For public and social AMS‐related activities, we searched Baidu (information) (https://www.baidu.com) (the main public search engine in China), and Chinese keywords were used, which included “antimicrobial/antibiotic resistance” OR “antimicrobial/antibiotic use,” AND “surveillance,” “antimicrobial stewardship,” “infection prevention and control,” “education,” “public awareness,” “medicines regulation,” “rational use,” “management/administration,” “propaganda,” “training,” “continuing education,” “meeting/conference,” and “ceremony.”

### 2.2 Evaluation

Following the Periodic National and Health‐Care Facility Assessment Tools in WHO policy guidance on integrated AMS activities, the implementation of AMS in China was evaluated item by item with five categories: No (the core element is not in place and is not a priority, 0 score); No, but a priority (the core element is a priority but there is no plan in place to initiate it, 1 score); Planned but not started (the core element is planned but no action has taken place, 2 score); Partially implemented (the core element is in place, but it is only partially implemented requiring further strengthening, 3 score), and Fully implemented (the core element is in place and is fully implemented without requiring strengthening but needing to be sustained, 4 score). Two investigators conducted the evaluation collaboratively, a discussion result will be reached if there was a disparity [9].

## 3 RESULTS

### 3.1 Data retrieval and search

**Figure 1:**
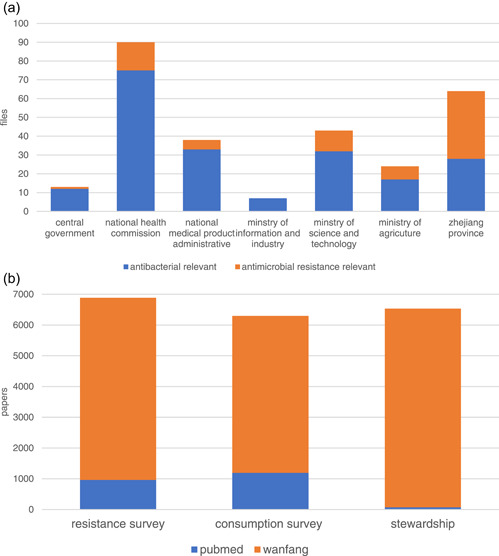
Retrieval and search results of the AMS relevant data. (a) Governmental files from the website of central and ministerial webpages. (b) Scientific publications from PubMed and Wanfang data.

By searching the Chinese central government (http://www.gov.cn), NHC (http://www.nhc.gov.cn), Ministry of Science and Technology (MOST) (http://www.most.gov.cn/index.html), Ministry of Agriculture (MOA) (http://www.moa.gov.cn), Ministry of Industry and Information Technology (MIIT) (http://www.miit.gov.cn), and National Medical Product Administration (NMPA) (http://www.nmpa.gov.cn) websites, 176 documents related to antibiotics, and 39 documents related to AMR were obtained. Zhejiang provincial government affairs website (http://www.zjzwfw.gov.cn) contained 28 documents related to antibiotics and 36 documents related to AMR (Figure 1).

In the search for scientific publications on antimicrobial use, bacterial resistance surveillance, and AMS in China from 2011 to 2021, there were 1194, 962, and 73 papers in PubMed and 5104, 5925, and 6461 papers in Wanfang data, respectively (Figure 1).

The output obtained from Baidu comprehensive search is too large to screen, so we limited the scope of the search to the subsection of “encyclopedia information.” There were 124, 169, and 155 pieces of information about antibiotics, bacterial resistance, and AMS, respectively. However, most of them are the forwarding of government documents on different web pages and there was a lack of information about public education or activities on bacterial resistance and rational use of antibiotics.

### 3.2 Evaluation of the integrated AMS item by item (Table 1)

**Table 1:** Evaluation of the integrated AMS activities of China by the criteria of WHO

Indicators	Status	Major actions	Liable bodies	Comments
**Pillar 1: Establish and develop national coordination mechanisms for antimicrobial stewardship and develop guidelines**				
*1. Establish and maintain a national coordinating mechanism for AMS, which is functional at the national, subnational, and district levels*				
1.1 Is there a national policy on integrated AMS activities?	5	NAP (2016–2020) was issued in 2016.	Leading by NHC and integrating another 13 ministries.	The purposes, actions, and responsibilities being clarified to each entity.
1.2 Is there a central national coordination unit at the ministry of health or designated agency or institution focused on AMS?	4	Multiministry coordinating mechanisms in AMR containment are being set up.	The NHC convenes the annual meeting to discuss AMR containment strategies and coordinates the overall action.	There were some briefings about the annual meeting, but without full information, such as strategies and actions, a regular report about the implementation being needed.
1.3 Has the central national coordination unit established a national coordination mechanism for integrated AMS activities with a diverse membership, including civil society and the private sector?	4	NHC and MOA set up several working groups for surveillance and education (e.g., NHC: AMR and AMU surveillance evaluation committee, an expert committee for drug rational use. MOA: Expert committee for animal drug residues and AMR).	Several working groups about surveillance and education being set up (referring to the left column). Some professional societies such as CMA, CMDA, and CVMA being involved.	No private entity or NGO participation in the mechanisms.
1.4 Have similar coordination mechanisms with diverse members, including from civil society and the private sector, being established at the subnational and district levels?	3	Few subnational coordination mechanisms being set up at the provincial level. Implementing NAP and promoting AMR containment action locally.	Most of the coordinators being provincial health authorities.	
1.5 Are there clear reporting lines and feedback mechanisms from subnational mechanisms to the national coordination mechanism on integrated AMS activities?	0			A closer relationship between national and subnational mechanisms being encouraged.
1.6 Is there funding allocated for the national coordinating unit and the national, subnational, and district coordination mechanisms?	0			No report or information was released about funding allocation.
1.7 Does the national coordination unit have clear terms of reference (TOR)?	4	NAP specified the TOR to each ministry.		More detailed responsibilities being not disclosed.
1.8 Is there a linkage to other relevant stakeholders, e.g., from tuberculosis (TB), infection protection and control (IPC), water, sanitation and hygiene (WASH) or universal health coverage (UHC)?	3	Yearly protocol from NHC about AMR containment and special campaign including IPC and UHC. From 2021 on, changing the concept of AMR from antibacterial resistance to antimicrobial resistance.	NHC	WASH being not involved in the NAP, but most of the requirements being integrated into the other regulations or rules for health‐care facility management and administration.
1.9 Is there a monitoring and evaluation (M&E) framework and have national targets been set for AMS activities based on nationally and internationally agreed indicators?	3	A national committee is being set up for evaluating the implementation of NAP.	NHC	Evaluating by the NAP rather than the indicators.
1.10 Have other programs, such as IPC, WASH, TB, malaria, HIV, UHC, and primary health care (PHC), integrated AMS activities within their action plans?	3	TB, HIV, and malaria programs being involved, and AMR control being being promoted in the PHC setting.	NHC, CCDC	CCDC taking some actions in eradicating TB, HIV, and malaria, including population screening and free treatment.
*2. Develop national treatment and stewardship guidelines, standards, and implementation tools*				
2.1 Have the national treatment guidelines for the management of infections been updated within the last 3–5 years?	4	NGA being issued in 2012 and revised in 2017 and 2022; PCUA being issued in 2005 and revised in 2015.	Both the guidelines are being supervised and promoted by the NHC.	Considering the wide territory and large difference in clinical practice and AMR prevalence, more education and promotion and localization of guidelines should be conducted.
2.2 Do the national treatment guidelines include AMS principles?	3	AMS concept being included in the PCUA.	NHC	Some more AMS strategies should be introduced and concretized in professionals.
2.3 Is there monitoring of implementation and compliance with treatment guidelines?	3	The NSAC contains the evaluation of compliance with guidelines.	NSAC	All the institutes should conduct the monitoring of guideline compliance.
2.4 Is there coordinated guidance and interventions to improve availability and appropriate use of diagnostics to guide therapeutic decisions?	1	No diagnostic guideline.	NHC, the yearly AMS protocol contains some articles to promote the microbiological test for definitive therapy.	The coordination or interventions to improve the appropriate use of diagnostics should be addressed.
2.5 Are there specific standard operating procedures for AMS activities in health‐care facilities and in community settings?	0	No standard operating procedures being issued.	NHC and all health‐care institutes.	The SOPs for the AMS actions should be generated soon.
2.6 Are there mechanisms and activities for the dissemination of guidelines, standards, and implementation tools on AMS activities?	1	Some education is conducted by societies, most of which being continuing medical education.	NHC, hospitals, and societies.	A top‐down national mechanism for the promotion of AMS should be set up.
**Pillar 2: Ensure access to and regulation of antimicrobials**					
*3. Improve access to essential, quality‐assured, safe, effective, and affordable antimicrobials*					
3.1 Has the WHO Model List of essential medicines (EML) and Access, Watch, Reserve (AWaRe) system been incorporated into the national EML formulary and health‐care facility treatment guidelines?	3	The NEML is issued and revised regularly. All hospitals are required to prescribe antibiotics in NEML to some percentages. Antibiotics formulary restriction policy being implemented in all hospitals, the antibiotics categorization lists being generated by each provincial health‐care authorities.	NHC, PHC	More strategies to promote the implementation of AWaRe should be established.	
3.2 Is there a system in place to monitor access to essential, quality‐assured, safe, effective, and affordable antimicrobials?	3	NMPA encouraging the consistency evaluation of generic antibiotics and the NHSA promoting and monitoring the use of the affordable agents.	NMPA, NHSA	The innovative antimicrobials against AMR germs should be easily access, for example, accelerating registration and insurance cover.	
3.3 Is there a system to periodically identify the availability of affordable antibiotics at health‐care facilities?	4		NHC, NMPA	The supply of antibiotics in hospitals was adequate.	
3.4 Is there a mechanism in place to report shortages and stock‐outs of antibiotics in the country?	4		NHC, NMPA	The supply of antibiotics in hospitals was adequate.	
3.5 Is there a mechanism to report the antibiotics used by patients?	3	NSNC monitoring the use in secondary and tertiary hospitals, but no monitoring of patient use in pharmacy and primary institutes.	NHC, NMPA	Antibiotics use monitoring in primary health‐care setting and sale in the pharmacy should be conducted.	
3.6 Is there a process to report the antibiotics used in the AWaRe system?	0	NSNC surveys by its own category list, not the AWaRe.	NHC	The integration of the surveillance category list with AWaRe being valuable.	
*4. Regulate social triggers and remuneration policies that promote responsible antimicrobial prescription and dispensing behaviors*					
4.1 Are health worker behavioral change principles incorporated into policies addressing diagnosis, prescription, dispensing, and administration of antimicrobials?	3	ARCU containing the articles to regulate prescribers’ behaviors; the health‐care quality indicators for hospital containing the requirements.	NHC, NSNC	Some professional strategies to change the behaviors of prescribers should be addressed.
*5. Legislate and regulate responsible and appropriate use and disposal of antimicrobials*					
5.1 Is there a regulation on prescription‐only sale/dispensing of antibiotics?	4	DAA including the articles about dispensing antibiotics by the prescription only.	All hospitals and pharmacies. NMPA	
5.2 Are regulations on dispensing antibiotics by prescription only being enforced (where access is not an issue)?	3			The phenomena of antibiotics dispensing without prescription existing in some rural areas.
5.3 Is there an enforceable regulatory mechanism to prohibit the sale of substandard and falsified drugs?	4	DAA	NMPA	
5.4 Are there standards and criteria for responsible manufacturing and disposal of antimicrobial agents?	4	GMP and GSP issued by NMPA.	NMPA	
**Pillar 3: Improve awareness, education, and training**				
*6. Improve awareness and engagement to support the behavioral change of antimicrobials use*				
6.1 Have studies on determinants of behavior in health professionals and other stakeholders, including the general public, been completed to support design of awareness campaigns?	3	Some nongovernmental studies were conducted, most of which were conducted by professional entities.	NHC, NMAP, and professional.	Nonsystematical investigation being conducted, only having local or small‐scale studies, and without consecutive investigations to follow up
6.2 Does the country hold World Antimicrobial Awareness Week (WAAW) activities annually?	4	Yearly ceremony organized by NHC	NHC, hospitals, and mass media.	More and more hospitals holding annual activities during WAAW, most of them locating in large cities.
6.3 Does the country have regular public awareness campaigns on the responsible and appropriate use of antibiotics?	0		NHC, MCT, NRTA	Raising the public awareness to AMR being urgent priority.
6.4 Are tailored AMS messages integrated into broader health promotion, prevention, treatment and rehabilitation services, and initiatives such as World Water Day, World Toilet Day, World Children's Day, immunization campaigns, World AIDS Day, World TB Day, World Malaria Day, and World Nutrition Week for sustained action?	0	NAP containing some articles about the broader health promotion.	NHC, NMPA, CCDC, MOHURD, MEE	
6.5 Is regular assessment/evaluation of the impact of education and awareness campaigns on knowledge attitudes and behaviors of health workers and the public conducted?	0	Some pilot investigations conducted by professional investigators	NHC, NRTA, MCT	Without regular actions.
*7. Strengthen health worker capacity through the provision of tailored education and training packages according to health worker roles and functions*				
7.1 Are AMS principles and strategies included in the educational curriculum of preservice health‐care professionals?	0	NAP content	MOE, NHC	
7.2 Is there access to in‐service training, including continuous professional development (CPD) on antimicrobial prescribing and AMS for all health‐care professional groups in the country?	3	NAP and ARCU	NHC, hospitals, and societies.	Some Pharm involving in the education and the educating hours being not enough.
7.3 Are AMS concepts and principles incorporated within the curriculum of other complementary disciplines e.g. the curriculum for IPC professionals?	0		MOE	There is no graduate curriculum of IPC now.
**Pillar 4: Strengthen water, sanitation, and hygiene and infection prevention and control**				
*8. Enhance WASH in health facilities and communities*				
8.1 Is there representation of the AMS coordinating unit on the WASH programs and vice versa?	1	All these were included in the mandatory standards for hospital construction and water supply; getting rid of poverty plan; all cities and part of rural area providing tap water.	NHC, MOHURD, MOA	
8.2 Is the WHO WASH Facility Improvement Tool (FIT) being promoted to assess WASH in health‐care facilities?	3			
*9. Implement IPC core components in health facilities*				
9.1 Is there representation of the AMS coordinating unit on the IPC programs and vice versa?	3	NAP contents, ARHI.	NHC and hospitals.	
9.2 Are there systems linking the monitoring and reporting of health‐care‐associated infections (HAIs), antimicrobial use, AMR, patient outcomes and quality of care?	3	Yearly concise report of the surveillances.	NHC	No integrating analysis, such as the association, risk factors, etc.
**Pillar 5: Surveillance, monitoring, and evaluation**				
*10. Surveillance of antimicrobial use and consumption*				
10.1 Is there a national surveillance program for AMR, consumption, and use with defined structures, governance, and work objectives (i.e., data collection, validation, analysis, reporting and data sharing with all stakeholders)?	3	NSNC and NSNR using indicators recommended by WHO, covering more than 2000 hospitals; some data feeding back to provincial authorities.	NHC and hospitals.	Without primary health‐care data; data sharing not being enough; not by inhabitant population indicators.
10.2 Are there guidelines/standard operating procedures for the use of (a) antimicrobial medicines use (AMU), (b) antimicrobial medicines consumption (AMC) and (c) AMR data to inform action for national and institutional decision‐making?	3	Yearly briefing about AMR and antibiotics use from 2017 on. Data being disclosed in part. NHC making use of the data to set AMS protocol.	NHC and hospitals.	No guideline or standard operating procedure for all these.
*11. Surveillance of AMR*				
11.1 Is there a plan to strengthen laboratory capacity to support accurate diagnosis for decision‐making?	3	Yearly protocol of AMS and PCUA addressing it. Health‐care quality indicators including this.	NHC and hospitals.	No consolidated measures.
*12. Monitoring and evaluation of AMS activities*				
12.1 Is there a national M&E framework, including internationally and locally agreed key performance indicators for integrated AMS interventions and activities in human health?	0		NHC	
12.2 Is there an accountability mechanism put in place at the local level which includes positive feedback and recognition of local leadership?	0		NHC, PHC	

Abbreviations: AMS, antimicrobial stewardship; ARCU, administrative regulations for the clinical use of antibiotics; ARHI, administrative regulations for hospital infections; CCDC, Chinese Center for Disease Control and Prevention; CMA, Chinese Medical Association; CMDA, Chinese Medical Doctor Association; CVMA, Chinese Veterinary Medical Association; DAA, Drug Administrative Act; GMP, good manufacture practice; GSP, good sale practice; MCT, Ministry of Culture and Tourism; MEE, Ministry of Ecology and Environment; MOA, Ministry of Agriculture; MOE, Ministry of Education; MOHURD, Ministry of Housing and Urban–Rural Development; NAP, National Action Plan for the Containment of AMR; NEML, National Essential Medicine List; NGA, National Guideline for Antimicrobial Therapy; NHC, National Health Commission; NHSA, National Healthcare Security Administration; NMPA, National Medical Product Administration; NRTA, National Radio and Television Administration; NSNC, National Surveillance Network for the Consumption of Antibiotics in Hospital (called as CASN); NSNR, National Surveillance Network for Bacterial Resistance (called as CARSS); PCUA, principles for clinical use of antimicrobial agents; PHC, Provincial Health Commission; TB, tuberculosis (TB), (IPC), water, sanitation and hygiene (WASH), or universal health coverage (UHCTOR), terms of reference.

#### 3.2.1 Establishment and development of national coordination mechanisms for AMS and development guidelines

A relatively comprehensive integrated AMS system has been established at the national level. The landmark event is the issuance of the “NAP to Contain AMR (2016–2020)” in 2016. The plan was jointly formulated by 14 ministerial governmental entities, and the NAP put forward clear 5‐year goals. It covers the research and development, approval and registration, production and circulation, use and other links of antibiotics, and also includes the participation of relevant departments of professionals and the public. The involvement of the Ministry of Finance should be a guarantee of resources to implement the plan. The responsibilities of each participating ministry are clarified and their objectives are aligned. The plan fully embodies the concept of One Health, integrates the health, agriculture, and environmental protection departments, takes corresponding actions on all links of the generation and transmission of AMR, strengthens the management of antibiotics use in healthcare and agriculture, and puts forward measures to deal with possible environmental hazards caused by antibiotics. After the launch of the NAP, the NHC served as the coordinating role among multiple sectors for the implementation of the plan and held a working meeting annually to discuss the strategies of AMR containment (Table 1, items 1.1, 1.2). Under the framework of the plan and multisectoral coordination mechanism, professional societies and advisory groups have been established in the field of health and agriculture to carry out scientific research and consultation on AMS (Table 1, item 1.3) [8, 10, 11, 12]. Since 2021, the NHC has changed the concept of antibacterial resistance to AMR, which means the resistance to tuberculosis, human immunodeficiency virus, malaria, and so forth was enclosed (Table 1, item 1.8) [13]. At the same time, the NHC has mandatorily implemented the national AMS in healthcare institutions, and regularly promulgates the annual AMS rules and main contents every year. The main indicators for healthcare institutes are the prescription rate and use intensity of antibiotics. Unfortunately, up to now, there is a lack of systematic AMS guidelines in detail in line with international standards such as the United States and the United Kingdom, and there are no regular evaluation and measurement of AMS implementations [6].

The NHC promulgated the “principles for clinical use of antimicrobial agents (PCUA)” in 2005 and revised it in 2015. The PCUA mainly include the basic principles of rational use of antibiotics, the basic concept of AMS, the major characteristics of main antibiotics, and the recommendations of antibacterial chemotherapy for common infections. In 2012, the “National Guideline for Antimicrobial Therapy (NGA)” was formulated, which was published in the form of a pocket manual, including empiric and definitive chemotherapeutic choice of infections, pharmacological characteristics of antibiotics, application of antibiotics in pediatrics, and the adverse reactions of antibiotics (Table 1, item 2.1) [14].

Unfortunately, there are no AMS guidelines or tools developed by the NHC or any other communities, nor are there relevant budget reports on the fund allocation to AMS (Table 1, item 1.6). At the subnational and district levels, through searching Baidu and Zhejiang provincial government websites, no multidepartmental coordination mechanism was found, and all provinces and regions mainly carry out AMS in accordance with national requirements and administrative regulations. The interaction and linkage between national and subnational AMS focal points were weak (Table 1, items 1.4, 1.5).

#### 3.2.2 Ensuring access to and regulation of antimicrobials

China has established an essential medicine system, and formulated and promulgated the essential medicine lists (EMLs) based on the WHO model list of essential medicine. The latest version of EML was issued in 2018, but antibacterial agents were not classified according to the Access, Watch, Reserve (AWaRe) system of WHO recommended in 2017. For the implementation of essential medicine policy, medical institutions are required to use a certain proportion of the essential medicines with enforcement of some relevant regulations (Table 1, items 3.1, 3.6) [15]. There is no drug shortage phenomenon in China. Due to the existence of many generic pharmaceutical enterprises to manufacture drugs with expired patents, the supply of essential drugs is very sufficient, and the main goal of the drug administration is to ensure the quality of generic drugs. The NMPA has put forward requirements and standards for conducting consistency evaluations on generic drugs. After satisfying the consistency standards, generic drugs could be included in the centralized procurement catalog for healthcare insurance [16, 17]. The NHC has established a surveillance network for the use of antibiotics in medical institutions in 2005 (Table 1, items 3.6, 10.1) [18]. The NMPA has established a drug sales monitoring network to survey the drug sales throughout the country; however, the survey on the use of antibiotics in pharmacies and communities was absent.

There are systematic laws and regulations for the registration, production, and sale of antibiotics. Antibiotics being prescription drugs have been clearly described in the “Drug Administration Act.” The law stipulates that drugs are divided into prescription and over‐the‐counter drugs. All antibiotics belong to prescription drugs and must be sold and dispensed by prescriptions issued by physicians [19]. The articles of the law are gradually being implemented. In large and medium‐sized cities, the sale of antibiotics by prescription performed well, but in contrast, the phenomena of over‐the‐counter sales of antibiotics in some rural and small towns are common (Table 1, item 5) [20].

Another core regulation is the “ARCU” issued by the NHC in 2012, which basically integrates the international AMS concept and strategies, intending to develop a comprehensive AMS system, including team building, infrastructures, professionals, management objectives, and legal responsibilities, for medical institutions. The ARCU is a critical sign of the implementation of AMS across the country. Focusing on the objectives of the regulation, the NHC formulates and promulgates AMS protocols every year to promote AMS in medical institutions across the country (Table 1, items 1.8, 4.1) [7].

#### 3.2.3 Improving awareness, education, and training

In the launching ceremony of the World Antibiotic Awareness Week (WAAW) in November every year, the NHC has some activities in individual regions and medical institutions [21]. But AMR containment was not involved in other relevant WHO events such as World AIDS Day, World TB Day, World Malaria Day, and World Nutrition Week. There is a lack of publicity and public education on the rational use of antibiotics and AMR control among the general public, no national public activity related to AMR has been launched, and there is no public education and activity introduced by the mass media (television, radio, newspapers, internet, etc.). Most AMS education and training sessions are organized by relevant professional communities and associations and are continuing with giving education to healthcare professionals in secondary and tertiary hospitals. The training and education of primary medical personnel are very insufficient. Although it is clearly stipulated in the ARCU that prescribers need AMS training before antibiotics prescription accreditation, maintenance, and upgrading, the overall implementation is not very ideal and the training courses are insufficient (Table 1, item 6) [22].

There is no curriculum of “pharmacotherapy” in the Chinese medical education system, no professional course in AMS, and no graduate major in infection control in medical colleges and universities.

#### 3.2.4 Strengthening water, sanitation and hygiene, and infection prevention and control

Over the past 10 years, Chinese medical and healthcare facilities have made great progress. A lot of large modern hospitals have been constructed around the country. The relevant WASH content has become a mandatory standard for the construction of hospitals, and the construction of primary medical institutions has basically been invested by the government and conforms to relevant national standards including “Code for Architectural Design of General Hospitals (2014)” and “Guidelines for Hospital Construction in China (2015)” (Table 1, items 8.1, 8.2) [23].

All healthcare institutions have set up various quality management committees such as the hospital infection prevention and control (IPC) committee, pharmaceutical committee, and so forth. These committees are mandatory requirements for medical institutions stipulated by laws and regulations and are mostly composed of multidisciplinary personnel, including administrative personnel, clinicians, nurses, logistics personnel, and so forth. Pharmaceutical experts are often included in the IPC committee, and IPC members are also included in the pharmaceutical committee. According to this investigation, the IPC department is the major focal point in the implementation of AMS in hospitals [24]. The NHC has promulgated relevant IPC regulations, including “administrative regulations of hospital infection (ARHI)” and “administrative regulations of disinfection” to strengthen IPC work. Since 2016, the NHC has edited and published integrated reports on the surveillance of AMR, antibiotics use, and nosocomial infections, but there is a lack of relevant correlation analysis in depth (e.g., the relationship between AMR and AMU) (Table 1, item 9) [25].

#### 3.2.5 Implementation of surveillance, monitoring, and evaluation

In 2005, the NHC established a national surveillance network for AMR and antimicrobial use in medical institutions (called CARSS and CASN, respectively). At launch, the network covered only some 100 large tertiary hospitals, and the number of pilot hospitals increased gradually; by 2021, the networks had covered more than 2000 hospitals including both tertiary and secondary hospitals. In 2021, the NHC further required all secondary and tertiary hospitals to submit data to the networks [13, 18]. The surveillance report was partially disclosed in 2016, but lacked general details, especially on surveillance of antibiotics use. The AMR surveillance network mainly adopts the method of passive monitoring, and collects the routine clinical microbiological examination data of the hospitals quarterly for integrated analysis, without information about the type of infection and patient data. The main indicators of antibiotics use surveillance include the prescription rate and use intensity of antibiotics, the prophylaxis use of antibiotics in surgical procedures, and the proportion of antibiotics used by ATC categories (J01), and so forth, the classification system of WHO AWaRe has not been adopted. Due to the limitation of the surveillance protocol, there is no outpatient, primary healthcare institution and community data in the AMR network, and there is a lack of primary and community data on the use of antibiotics; no reference laboratory for AMR surveillance has been established (Table 1, items 10, 11). In addition, there are other AMR surveillance networks established by academic institutions, such as Chinet of Fudan University and BRICS of Zhejiang University.

At present, AMS implementation is mainly promoted at the national level, and subnational and district institutes mainly follow the national requirements. There is no specific AMS strategy to adapt to the actual situation in different areas and institutions, and there is basically no evaluation of the implementation and effectiveness of AMS (Table 1, item 12).

### 3.3 Overall evaluation of the integrated AMS at different levels

**Figure 2:**
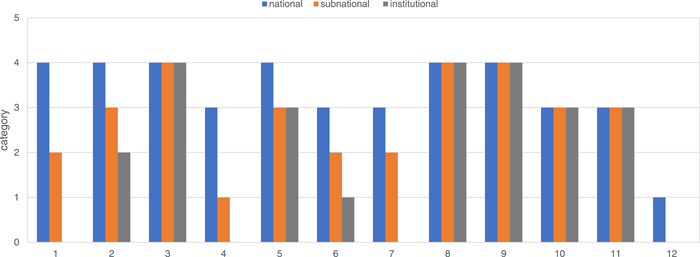
Evaluation of the integrative AMS system at the national, subnational, and district levels by a category scale recommended by the World Health Organization. The numbers on *X*‐axis mean: 1. Establish and maintain a national coordinating mechanism for AMS that is functional at the national, subnational, and district levels. 2. Develop national treatment and stewardship guidelines, standards, and implementation tools. 3. Improve access to essential, quality‐assured, safe, effective, and affordable antimicrobials. 4. Regulate social triggers and remuneration policies that promote responsible antimicrobial prescription and dispensing behaviors. 5. Legislate and regulate responsible and appropriate use and disposal of antimicrobials. 6. Improve awareness and engagement to support behavioral change of antimicrobials use. 7. Strengthen health worker capacity through the provision of tailored education and training packages according to health worker roles and functions. 8. Enhance WASH in health facilities and communities. 9. Implement IPC core components in health facilities. 10. Surveillance of antimicrobial use and consumption. 11. Surveillance of AMR. 12. Monitoring and evaluation of AMS activities. AMS, antimicrobial stewardship.

From the perspective of AMS implementation at different levels, a comprehensive AMS system has been established at the national level, including laws and regulations, administrative entities, coordination mechanism, infrastructure, guidelines and protocols, and the national AMS implementation has been continuously promoted through the annual AMS protocol. However, at the subnational and district levels, AMS has not yet formed a sustainable working mechanism, lack of multisectoral coordination entities and mechanisms, lack of social activities of AMR, lack of publicity and education to the public, and lack of continuous professional capacity‐building planning in healthcare institutions. There is a lack of national professional entities for the promotion of AMS, the evaluation and measurement of the implementation of AMS, and the surveillance of antimicrobial use and AMR in primary institutions and communities (Figure 2).

## 4 DISCUSSION

The Chinese government takes high priority to the control of AMR and has established a better legal system, multisectoral coordination mechanism, and surveillance system at the national level. The NHC jointing with other 13 ministries issued the NAP in 2016, the plan with comprehensive coverage integrating with the concept of “One Health” in AMR control emphasized the major strategies such as establishing multisectoral mechanisms in the prevention and control of AMR with clarification of each sectoral responsibility, providing a financial guarantee to the implementation of the plan, optimizing antibiotics consumption and resistance surveillance systems, and improving the capacity of professional personals in bacterial resistance prevention and control, and so forth [8, 26]. After the issue of the plan, a multiministerial mechanism was set up and the NHC took the coordination role in the implementation, there was an annual meeting to discuss the major objectives and actions [27]. Some regulatory files issued by the NHC, which included “Rules for pharmaceutical affairs in healthcare institutions” in 2002, ARHI in 2006 [28], “administrative regulations for prescription” in 2007 [29], ARCU in 2012 [7], could be supplementary policies for the better implementation of the plan.

From 2004 on, the NHC issued the PCUA (2004, revised in 2015) [14], National Formulary & National Formulary (Pediatric Edition) (2009, 2012), National Essential Drugs List (2009 Elementary Edition & 2012, 2018) [12], and the NGA (2012, revised in 2017) [30].

In 2005, the NHC established hospital antibiotic consumption surveillance and AMR surveillance networks to inspect the prevalence of bacterial resistance and the use of antibacterial in healthcare institutions. Up to now, the member hospitals in the networks have expanded to more than 2000 [18]. In 2021, the NHC called for all the secondary and tertiary hospitals in China to join the network and submit the data to the surveys [13]. However, the existing networks mainly cover large hospitals, and there is a lack of data from outpatient, community and primary institutions. The protocol of the AMR network consistently adopts passive surveillance. There is no survey based on patients or infections, no reference laboratory is available, and no data are submitted to WHO AMR surveillance (GLASS), which is helpful to further improve the quality and participate in international AMR control cooperation. The AWaRe classification system of antibiotics is not used in the monitoring of antibiotics use nor does it include the consumption in primary institutions, sales in pharmacies, and the use in a community. The main indicators are the DDDs of 100 inpatient‐day. The DDDs of 100,000 inhabitants were not employed in the survey, and this makes it impossible to directly compare with the data of other country networks.

In recent years, the National Institute of Hospital Administration, a subsidiary facility of the NHC, has led training programs and continuing education courses in rational antibiotics use for clinical pharmacists, infectious clinicians, and clinical microbiologists. Infectious disease physician and clinical pharmacists training was also conducted at the national and hospital level [7, 22, 30]. Compared with the huge hospital amount, the population of clinical pharmacists and infectious physicians are in the status of absolute shortage [31].

More importantly, in 2011, the NHC launched a national campaign for AMS in healthcare institutes [6]. Considering international successes in fostering the rational use of antibiotics, the government used antibiotic formulary restriction as a core strategy, set management targets, conducted education and training, and recommended strategies to hospitals. The NHC then conducted supervision and inspection to push the campaign forward by the end of each year, and any medical institutions and administrators, physicians, and pharmacists who violated the regulations or failed to meet targets were penalized. The special campaign laid the foundation for establishing the sustainable development of AMS, which is ongoing from 2011 forward [10]. By the year 2016, the antibiotics procurement expenditure in hospitals declined from 22.3% in 2010 to 12.1% in 2016 in total drug purchases. The antibacterial drugs prescribing rate for inpatients, outpatient, and the antibiotics prophylaxis rate in clean surgical incision significantly decreased during the past 6 years, which were 67.8%, 19.5%, and 97.9%, respectively, in 2010 and 40.8%, 8.5%, and 38.3% in 2016. The antibiotics utilization by defined daily dosage per 100 patient days (DDDs/100 patient days) was also reduced remarkably, which was 85.3 ± 29.8, 48.8 ± 7.7, and 48.5 ± 8.0 in 2010, 2013, and 2016, respectively [32].

Compared with the all‐around AMS system at the national level, the AMS system at the subnational and district levels still needs to do more. From the perspective of the existing healthcare management system, the provincial health authorities mainly focus on the implementation of various national AMS strategies and requirements. For example, all provinces have participated in the national surveillance networks, and have also carried out special campaigns of AMS since 2011. However, due to different population compositions and healthcare capacities in various regions, there are differences in the prevalence of drug resistance and the problems in the use of antibiotics, as well as in the fundamental challenges. All regions need to carry out targeted AMS strategies adapted to their own characteristics. So far, there is no provincial multisectoral coordination mechanism for AMR containment. For example, the heavy prevalence of carbapenem‐resistant *Enterobacteriales* in the developed regions needs to be curbed with more professional AMS strategies such as patient isolation and screening, but in the developing areas, the capacity building of professionals should be the key strategy for the superbug control [33, 34]. Ji et al. [34] conducted a survey about AMS among 1032 clinical pharmacists in Northwest China, the results showed that the pharmacists were interested in the actions of AMS, but there were some barriers to impede their participation, such as high workload (59.5% of respondents), ineffective communication between pharmacists and doctors (57.7%), and inadequate knowledge of AMS (47.0%).

The main AMS strategies in medical institutions should focus on professional interventions. The existing data did not find that medical institutions carry out continuous professional intervention for all links of drug resistance, such as the selective reporting of the clinical microbiological test, the consultation of infectious physicians, and the drug‐resistant bacteria eradication of infection control personnel [9]. Most hospitals have implemented the antibiotics formulary restriction in accordance with the ARCU. Doctors were accredited with different prescription rights for antibiotics at the professional level. Prescription reviews have been implemented in some hospitals, but the formulary restriction strategy in the overall medical institutions is more in the construction of the system, and there are still some challenges in the implementation. At the same time, the continuous medical education for prescription right maintenance did not meet the requirements. Zhou and Ma [33] investigated the AMS practice in 116 tertiary hospitals and found that 38.8% of hospitals coordinated AMS by medical service departments, 25.9% of hospitals did not have an infectious department, and 27.3% of hospitals could not perform the resistant epidemiological analysis in depth. Prescription preauthorization and preprescription review with feedback were implemented in only 50% of the hospitals. AMS and AMR control is a systematic project involving a wide range of relevant, which needs to be linked up and down and coordinated with each other. To achieve success in AMS implementation, the protocols of the management authorities need to be effectively implemented, and all actions of AMS need to be the conscious behaviors of relevant personnel. Healthcare institutions are the core unit of AMR control, whether medical institutions actively take responsibility is the key to the success or failure of resistance containment. Public awareness and active participation will ease the implementation of AMS and assist in the containment of AMR.

In summary, the Chinese government has established a system for AMS and implemented a multisectoral coordinative mechanism. The concept of rational use of antibiotics has been formed in the administrative system, even the guideline of COVID‐19 emphasized the use of antibiotics should strictly follow the indication of bacterial infections [35]. With the core documents of the ARCU and the NAP, a relatively comprehensive integrated AMS system construction, technical specifications, surveillance network, professional capacity building, and even education and training were in place. To some extent, the special campaign for AMS initiated by the NHC, being the main action and achieving certain results, which has laid the foundation for promoting AMS and AMR control in China, needs to be strengthened [36]. However, at the subnational and district levels, an urgent need to interact with the national AMS, implement the multisectoral AMS coordination mechanism, and carry out AMS practice based on professional interventions in medical institutions should be realized to build a pyramid‐like AMS system with upper and lower linkage. On the one hand, it is necessary to continuously implement government guidance in all hospitals, and on the other hand, a diversity of AMS strategies should be implemented in medical institutions, communities, and the public to converse the inverted pyramid of the existing AMS system.

## AUTHOR CONTRIBUTIONS

**Yonghong Xiao**: Conceptualization (equal); formal analysis (equal); funding acquisition (equal); investigation (equal); methodology (equal); project administration (equal).

## CONFLICT OF INTEREST

The authors declare no conflict of interest.

## ETHICS STATEMENT

None.

## INFORMED CONSENT

None.

## Data Availability

Data sharing is not applicable to this article as no new data were created or analyzed in this study.

## References

[1] The Review of Antimicrobial Resistance . Tackling drug resistant infections globally: final report and recommendations. 2016. https://amr-review.org/sites/default/files/160525_Final%20paper_with%20cover.pdf

[2] Antimicrobial Resistance Collaborators . Global burden of bacterial antimicrobial resistance in 2019: a systematic analysis. Lancet. 2022;399:629–55. 10.1016/S0140-6736(21)02724-0 35065702 PMC8841637

[3] WHO . Global action plan on antimicrobial resistance. 2015. Available from: https://www.who.int/publications/i/item/9789241509763

[4] https://www.un.org/pga/74/event/high-level-interactive-dialogue-on-antimicrobial-resistance/#:%7E:text=In%20September%202016,%20the%20UN%20General%20Assembly%20convened,meeting%20of%20the%20General%20Assembly%20on%20antimicrobial%20resistance

[5] WHO . Antimicrobial stewardship programmes in health‐care facilities in low‐ and middle‐income countries. A WHO practical toolkit. 2019. Available from: https://apps.who.int/iris/bitstream/handle/10665/329404/9789241515481-eng.pdf 10.1093/jacamr/dlz072 PMC8210188 34222945

[6] NHC . Notice of the General Office of the Ministry of Health on special campaign for the clinical use of antibiotics in China. 2011. Available from: http://www.nhc.gov.cn/cms-search/xxgk/getManuscriptXxgk.htm?id=51376

[7] NHC . Administrative regulations for the clinical use of antibiotics. 2012. http://www.nhc.gov.cn/wjw/c100022/202201/8fcae32c3f1f467eb795ee816e2387d6.shtml

[8] NHC . Notice on issuing the National Action Plan to Contain AMR (2016–2020). 2016. http://www.nhc.gov.cn/cms-search/xxgk/getManuscriptXxgk.htm?id=f1ed26a0c8774e1c8fc89dd481ec84d7

[9] WHO . WHO policy guidance on integrated antimicrobial stewardship activities. 2021. Available from: https://www.who.int/publications/i/item/9789240025530

[10] http://www.heliyongyao.org/fagui/

[11] http://kangnaiyao.mpf.org.cn

[12] http://www.gov.cn/xinwen/2017-05/03/content_5190681.htm

[13] NHC . Notice of the National Health Commission on Further Strengthening the management of antimicrobial drugs and curbing drug resistance. 2021. Available from: http://www.nhc.gov.cn/yzygj/s7659/202104/0a5f9d529e1d4f60a3f73abcb29410d0.shtml

[14] NHC . Notice on issuing the principles for clinical use of antimicrobial agents. 2015. Available from: http://www.nhc.gov.cn/yzygj/s3593/201508/c18e1014de6c45ed9f6f9d592b43db42.shtml

[15] NHC .National Essential Medicine List. 2018. Available from: http://www.nhc.gov.cn/wjw/jbywml/201810/600865149f4740eb8ebe729c426fb5d7.shtml

[16] The State Council .Opinions of the General Office of the State Council on the evaluation of the consistency of quality and efficacy of generic drugs. 2016. Available from: http://www.gov.cn/zhengce/content/2016-03/05/content_5049364.htm

[17] The State Council . A pilot program for centralized drug procurement and use. 2019. Available from: http://www.gov.cn/zhengce/content/2019-01/17/content_5358604.htm

[18] NHC . The establishment of bacterial drug resistance and clinical use of antibiotics surveillance network. 2005. Available from: http://www.nhc.gov.cn/wjw/zcjd/201304/b0da0ebfc7b3428f98d435a29cfa4250.shtml

[19] NPC Standing Committee . Drug Administrative Act. 2015. Available from: https://www.nmpa.gov.cn/yaopin/ypfgwj/ypflxzhfg/20150424120001309.html

[20] Gong Y , Jiang N , Chen Z , Wang J , Zhang J , Feng J , et al. Over‐the‐counter antibiotic sales in community and online pharmacies, China. Bull World Health Organ. 2020;98(7):449–57. 10.2471/BLT.19.242370 32742030 PMC7375218

[21] NHC .“World Antimicrobial Awareness Week” was officially launched. 2017. Available from: http://www.nhc.gov.cn/yzygj/jdt/201711/c3f7083ccb1d49798817e49dcebb1d99.shtml

[22] NIHA . The first Guangzhou training course for clinical pharmacists in the diagnosis and treatment of bacterial and fungal infections (Peiying program) opened. 2017. Available from: http://www.niha.org.cn/hwaciis/news/publish/sevenInner?id=401&title

[23] MOHURD . Announcement on issuing Code for Architectural Design of General Hospitals. 2014. Available from: https://www.mohurd.gov.cn/gongkai/fdzdgknr/tzgg/201412/20141209_224354.html

[24] NHC . Administrative regulations for healthcare quality. 2016. Available from: http://www.nhc.gov.cn/wjw/c100022/202201/922894b1072d4a8a91249407fea2471e.shtml

[25] NHC . The current situation of clinical application management and bacterial resistance of antibiotics in China was published. 2016. Available from: http://www.nhc.gov.cn/yzygj/s3594/201611/a2b9bca213a74ce88a21571795bdc13a.shtml

[26] Xiao Y , Li L . China's national plan to combat antimicrobial resistance. Lancet Infect Dis. 2016;16(11):1216–18. 10.1016/S1473-3099(16)30388-7 27788972

[27] NHC . The second meeting of the joint working mechanism for prevention and control of bacterial drug resistance was held in 2016. 2016. Available from: http://www.nhc.gov.cn/yzygj/s3594/201701/f6c25492531c41b9a9c30c073d5c15aa.shtml

[28] NHC . Administrative regulations of hospital infection. 2006. Available from: http://www.nhc.gov.cn/wjw/c100022/202201/22d85ce0b5f441d094538aff835c1aca.shtml

[29] NHC . Administrative regulations for prescription. 2007. Available from: http://www.nhc.gov.cn/fzs/s3576/201808/d71d4735f6c842158d2757fbaa553b80.shtml

[30] NHC . Notice of the General Office of the National Health and Family Planning Commission on further strengthening the management of clinical application of antibiotics and curbing bacterial resistance. 2017. Available from: http://www.nhc.gov.cn/yzygj/s7659/201703/d2f580480cef4ab1b976542b550f36cf.shtml

[31] Zhang C , Li S , Ji J , Shen P , Ying C , Li L , et al. The professional status of infectious disease physicians in China: a nationwide cross‐sectional survey. Clin Microbiol Infect. 2017;24(1):82.e5–82.e10. 10.1016/j.cmi.2017.05.008 28506783

[32] Xiao Y , Shen P , Zheng B , Zhou K , Luo Q , Li L . Change in antibiotic use in secondary and tertiary hospitals nationwide after a national antimicrobial stewardship campaign was launched in China, 2011–2016: an observational study. J Infect Dis. 2020;221(Suppl 2):S148–55. 10.1093/infdis/jiz556 32176788

[33] Zhou J , Ma X . A survey on antimicrobial stewardship in 116 tertiary hospitals in China. Clin Microbiol Infect. 2019;25(6):759.e9–e14. 10.1016/j.cmi.2018.09.005 30267932

[34] Ji W , Hayat K , Ye D , Mciver DJ , Yan K , Kadirhaz M , et al. Antimicrobial stewardship programs in Northwest China: a cross‐sectional survey of perceptions, involvement, and perceived barriers among hospital pharmacists. Front Pharmacol. 2021;12:616503. 10.3389/fphar.2021.616503 33995017 PMC8117155

[35] NHC . Guideline for the diagnosis and treatment of Covid‐19 (the ninth tentative version). 2022. Available from: http://www.nhc.gov.cn/yzygj/s7653p/202203/b74ade1ba4494583805a3d2e40093d88.shtml

[36] Xiao Y . Antimicrobial stewardship in China: systems, actions and future strategies. Clin Infect Dis. 2018;67(Suppl_2):S135–41. 10.1093/cid/ciy641 30423041

